# Multilevel Factors Impacting PrEP Engagement Among Young Gay Men and Young Transgender Women in Thailand: A Qualitative Analysis

**DOI:** 10.1177/23259582231188221

**Published:** 2023-07-17

**Authors:** Peter A. Newman, Suchon Tepjan, Kangwan Fongkaew, Pakorn Akkakanjanasupar, Jan Willem de Lind van Wijngaarden, Nuttapon Chonwanarat

**Affiliations:** 1 Factor-Inwentash Faculty of Social Work, University of Toronto, Toronto, Canada; 2 VOICES-Thailand Foundation, Chiang Mai, Thailand; 3 495258 Faculty of Humanities and Social Sciences, Burapha University, Chonburi, Thailand; 4 Independent Scholar/Consultant, Bangsaen Chonburi, Thailand

**Keywords:** HIV prevention, pre-exposure prophylaxis, adolescents, MSM, transgender youth, healthcare providers, qualitative research, Thailand

## Abstract

Half of new HIV infections in Thailand are among young people, the majority of whom are young gay and other men who have sex with men (YMSM) and young transgender women (YTGW). Amid low pre-exposure prophylaxis (PrEP) coverage, we explored practice-based factors that impact PrEP engagement among YMSM and YTGW. In 2018, we conducted 4 focus group discussions with 20 YMSM and 5 YTGW, and 22 in-depth interviews (5 in 2022) with healthcare providers (HCPs), community-based organization (CBO)/nongovernmental organization (NGO) staff, and peer educators. The inclusion of PrEP in universal healthcare coverage, including YMSM and YTGW, is a substantial facilitator of PrEP use; however, systemic barriers at microsocial (lack of communication about PrEP from HCPs, teachers, parents), mesosocial (healthcare-service fragmentation, lack of PrEP-competent HCPs), and macrosocial levels (annual quotas on free HIV-testing, HIV- and sexual-stigma) constrain and disincentivize adolescents’ engagement with PrEP. National scale-up of youth-friendly and LGBT-affirmative CBO/NGO clinics, HCP training, and tailored programs to support adolescents’ adherence may promote PrEP engagement among YMSM and YTGW in Thailand.

## Introduction

Pre-exposure prophylaxis (PrEP) is among the most promising biomedical developments in 4 decades of the AIDS epidemic. Nevertheless, low utilization of PrEP constrains its effectiveness in controlling the epidemic. Despite Thailand's earlier successes in averting a generalized epidemic,^1^ HIV prevalence remains high among gay and other men who have sex with men (MSM) and transgender women (TGW), especially young people. Overall, 50% of HIV incident infections in Thailand are among young people aged 15 to 24 years, the majority of these young MSM (YMSM).^2^ HIV prevalence among YMSM has been estimated at 7.0-7.2%,^2,3^ with HIV incidence reported at 10 per 100-person years among YMSM age 13 to 21 years, over 4 times higher than among MSM >30 years old.^4^ Higher HIV incidence has also been reported among young (≤21 years) transgender women (YTGW) than TGW >30 years old.^5,6^

Thailand has been the site of several PrEP clinical trials and demonstration projects since 2005, conducted largely with adult MSM and TGW.^1^ In 2014, the Thailand Ministry of Public Health^5,7^ recommended oral daily PrEP for high-risk individuals, specifically including MSM, available for self-pay and through demonstration projects. In October 2019, PrEP became reimbursable under the government's Universal Health Insurance Scheme, and was made available free of cost from physicians at government hospitals/clinics, private hospitals and clinics that join the national coverage scheme, and key population-led health services at community-based (CBOs) and nongovernmental organizations (NGOs), in addition to self-pay from other private clinics.^8,9^ As of October 2022, however, government regulations effectively barred civil society organizations from providing PrEP by failing to approve continued reimbursements for medical costs of HIV prevention services delivered by CBOs and NGOs, threatening to reverse progress in scaling up PrEP coverage.^10^

A 2021 Thai Ministry of Public Health report (p. 21),^8^ described “a gap requiring urgent expansion of PrEP coverage”: the 11,925 individuals from key populations who received PrEP as of 2021 under a government-supported free PrEP program represent only 8.3% of the identified target population. Earlier data from a large demonstration project of key population-delivered PrEP indicates that of 12,488 HIV-negative MSM and 2,463 HIV-negative TGW offered PrEP, 9.3% (n = 1,166) of MSM and 8.9% (n = 219) of TGW agreed to take PrEP.^11^ PrEP acceptance was lower among YMSM and YTGW ≤ 25 years than MSM and TGW > 25 years.^9^ In regard to young people, age-disaggregated data from 2017 indicate that adolescents comprised only 2% of PrEP users in Thailand,^12^ with clinical trials showing low adherence and high rates of PrEP discontinuation.^4^ Limited global PrEP trials with adolescents have demonstrated the feasibility and safety of oral PrEP use among at-risk youth, including YMSM, but similarly report adherence challenges and discontinuation due to an array of developmental and social factors.^13,14^ Increased focus is warranted on PrEP uptake and adherence in the contexts of young people's lives.^15^

International guidelines highlight the importance of location-population-specific approaches to effective HIV prevention program design, including meaningful involvement of key populations in PrEP implementation.^16–18^ Such approaches are crucial to differentiated service delivery of PrEP for young people,^12^ whose behaviors and opportunities are influenced by multiple domains of their social ecology, including healthcare settings, schools, families, and peers.^19,20^ While clinical trials and demonstration projects have provided valuable information about PrEP acceptability and implementation, they have been conducted predominantly with adults in the Global North^15^ and examine PrEP from a biomedical perspective—as a concrete tool to be added to existing combination prevention interventions.^21,22^ Scant research addresses PrEP use among adolescents in the Global South, and from the perspective of multilevel social and institutional practices that impact on their engagement with PrEP.

With unmitigated new HIV infections and low PrEP coverage among YMSM and YTGW in Thailand, and amid threats to expanded PrEP access, we aimed to explore and understand how gay and transgender young people's engagement with PrEP is impacted by multilevel factors and social practices across their social ecology.

## Methods

### Conceptual Framework

We used a practice-based combination prevention framework^22^ to guide our analysis. The framework foregrounds links between individual behaviors and structural factors rather than structural factors per se,^23^ such as how public health programs and policies, healthcare providers (HCPs) and institutional practices affect individual behaviors and broader social practices.^24^ The practice-based typology identifies material factors as tangible objects, technologies, and infrastructures, while motivational factors comprise desires and aspirations that affect PrEP engagement. Competence factors are associated with practice know-how about how, when, and where to engage with PrEP. Relational factors traverse individual identities and demographics, and interactions with HCPs, partners, peers, and institutional environments. Symbolic factors include PrEP acceptability and stigma among individuals, peers, partners, families, and social representations, cultural norms, and public discourse that affect young people's PrEP engagement.^^22^^

### Participants and Setting

Young gay men (YGM) and YTGW were recruited by a trained peer in high schools, vocational schools, and universities in 3 semiurban provinces east of Bangkok. The research team also contacted teachers who worked with school clubs and campus associations for lesbian, gay, bisexual, and transgender (LGBT+) students, who provided information about the study. All recruitment was conducted by word-of-mouth. Young people who were interested contacted a peer research assistant or study staff. Eligibility criteria were being 16 to 20 years of age and self-identified as gay or transgender.

Key informants (KIs) were selected using purposive sampling to comprise individuals with diverse roles–including HCPs (ie, nurses, physicians, and counselors), clinic and CBO/NGO staff/administrators, and peer educators, and varied settings. All were experienced in working with gay and transgender young people. KIs were contacted by publicly available phone or email addresses and invited to participate.

### Data Collection

Two members of the research team, 1 researcher and a master's student research assistant, co-facilitated 75 to 90 min Thai-language focus group discussions (FGDs) with young people from June to August 2018. FGDs were segmented by age (16-17 and 18-20 years old) to facilitate comfort and openness, particularly about HIV and sexuality, in a group setting.^25^ All members of the research team conducted semistructured, 45 to 60 min in-depth interviews with KIs, in Thai or English, based on participants’ preferences. KI interviews were conducted from June to August 2018 (n = 17), and from June to July 2022 (n = 5) to explore impacts of evolving government regulations regarding expanded PrEP coverage.

We developed a semistructured FGD topic guide based on a literature review,^14,26–28^ team members’ experiences in HIV prevention with young people in the Asia-Pacific region, and a social ecological model.^19,29^ Open-ended questions and probes explored knowledge, sources of information, attitudes, practices, and experiences regarding sexuality/gender identity, HIV, and PrEP, with questions posed about oneself or one's peers. Items included: Have you or your friends heard about PrEP or pre-exposure prophylaxis? What do you know about PrEP? From where did you get information? Have any of your friends used PrEP? What are some of the challenges facing gay or transgender youth in terms of HIV/risk reduction? Access to healthcare services? LGBT-friendly services? The KI interview guide mirrored the FGD topic guide to explore perspectives and experiences on working with YGM and YTGW as well as institutional, sociocultural, and policy factors that influence their engagement with PrEP.

### Data Analysis

FGDs and KI interviews were digitally recorded and transcribed in Thai or English, and reviewed for accuracy by the research team. Transcripts were uploaded to Atlas.ti (Scientific Software Development, Berlin) to support team-based data coding and analysis. After immersion in the transcripts, 3 researchers developed a codebook. The codebook was applied to the same transcripts by teams of 2 independent coders, with team meetings held to discuss discrepancies in coding and arrive at consensus. Transcripts were then coded independently using thematic analysis^30,31^ guided by a social ecological model.^19^ Transcripts were analyzed using deductive (applying the codebook) and inductive methods (identifying emergent codes) in an iterative process, with new codes added to the shared database.^32^ Data saturation was achieved as no new codes or concepts emerged in the final transcripts.^33^ In a previous analysis of these data, we focused on barriers to HIV testing among MSM and TGW adolescents.^32^ In the present analysis, also including additional KI interviews, we focused on PrEP and applied a practice-based HIV prevention framework^22^ to examine systemic challenges in healthcare practice and policy that impact on PrEP implementation for YMSM and YTGW.

### Ethical Approval and Informed Consent

Ethical approval was granted by the Institutional Review Board of Burapha University (Reference # Hu 024/2561) and the Health Sciences Research Ethics Board of the University of Toronto (Reference #35253). All participants provided written informed consent; adolescents <18 years old provided parental consent prior to enrollment in the study. To mitigate risks to young people who were not out to their parents, consent forms did not indicate being gay or transgender, and referred to adolescent health and wellbeing. Young people were compensated 300 Thai Baht (∼$10 US) and KIs 500 Thai Baht for their participation.

## Results

### Participant Characteristics

We conducted 4 FGDs (n = 5-7/group) with YGM (n = 20) and YTGW (n = 5), mean age = 18.0 years (SD = 1.3). KI interviews (N = 22) were conducted with HCPs (n = 6), hospital/clinic administrators and staff (n = 2), CBO/NGO administrators/staff (n = 5), peer counselors/educators (n = 6), public health officers (n = 2), and educators (n = 1) (see Table 1).

**Table 1.** Focus Group Discussion and Key Informant Demographics (N = 47).

FGD participants (n = 25)				
ID	N	Sexual orientation/gender identity	Age (years)	Organization type
**FGD-1**	6	1 TGW / 5 GM	18-20	University
**FGD-2**	6	2 TGW / 4 GM	16-17	Vocational and high school
**FGD-3**	6	1 TGW / 5 GM	16-17	High school
**FGD-4**	7	1 TGW / 6 GM	18-20	University
Key informants (KI) (n = 22)				
ID	Role			
**KI-1**	Admin., Director			CBO for transgender persons
**KI-2**	Admin., Director			CBO for key populations
**KI-3**	Educator, Lecturer/Researcher			University
**KI-4**	HCP, Nurse			Government hospital
**KI-5**	HCP, Nurse			University clinic
**KI-6**	HCP, Physician			Government hospital & sexual health clinic
**KI-7**	Admin., Co-Director			CBO for key populations
**KI-8**	HCP, Nurse			Government hospital
**KI-9**	PHO, Health Counselor			University clinic
**KI-10**	Peer Educator, Peer Education Expert			NGO for key populations
**KI-11**	HCP, Nurse			Hospital-based youth clinic
**KI-12**	PHO, Health Researcher/Educator			Government hospital
**KI-13**	Admin., PE Manager			NGO for young key populations
**KI-14**	Admin., Director			NGO for key populations
**KI-15**	Peer Educator, Peer Educator & Outreach			NGO for key populations
**KI-16**	Peer Educator, Transgender Student Council Member			University
**KI-17**	Peer Educator, Gay Student Council Member			University
**KI-18**	HCP, Nurse			Government sexual health clinic
**KI-19**	Admin., Supervisor			Government sexual health clinic
**KI-20**	Peer Counselor, Sexual & HIV Counselor			NGO for key populations
**KI-21**	Peer Counselor, Sexual & HIV Counselor			NGO for key populations
**KI-22**	Admin., Clinic Administrator			Government sexual health clinic

Abbreviations: Admin., administrator; CBO, community-based organization; FGD, focus group discussion; GM, gay man; HCP, healthcare provider; KI, key informants; NGO, nongovernmental organization; PHO, public health officer; TGW, transgender woman.

### Themes

**Figure 1.**
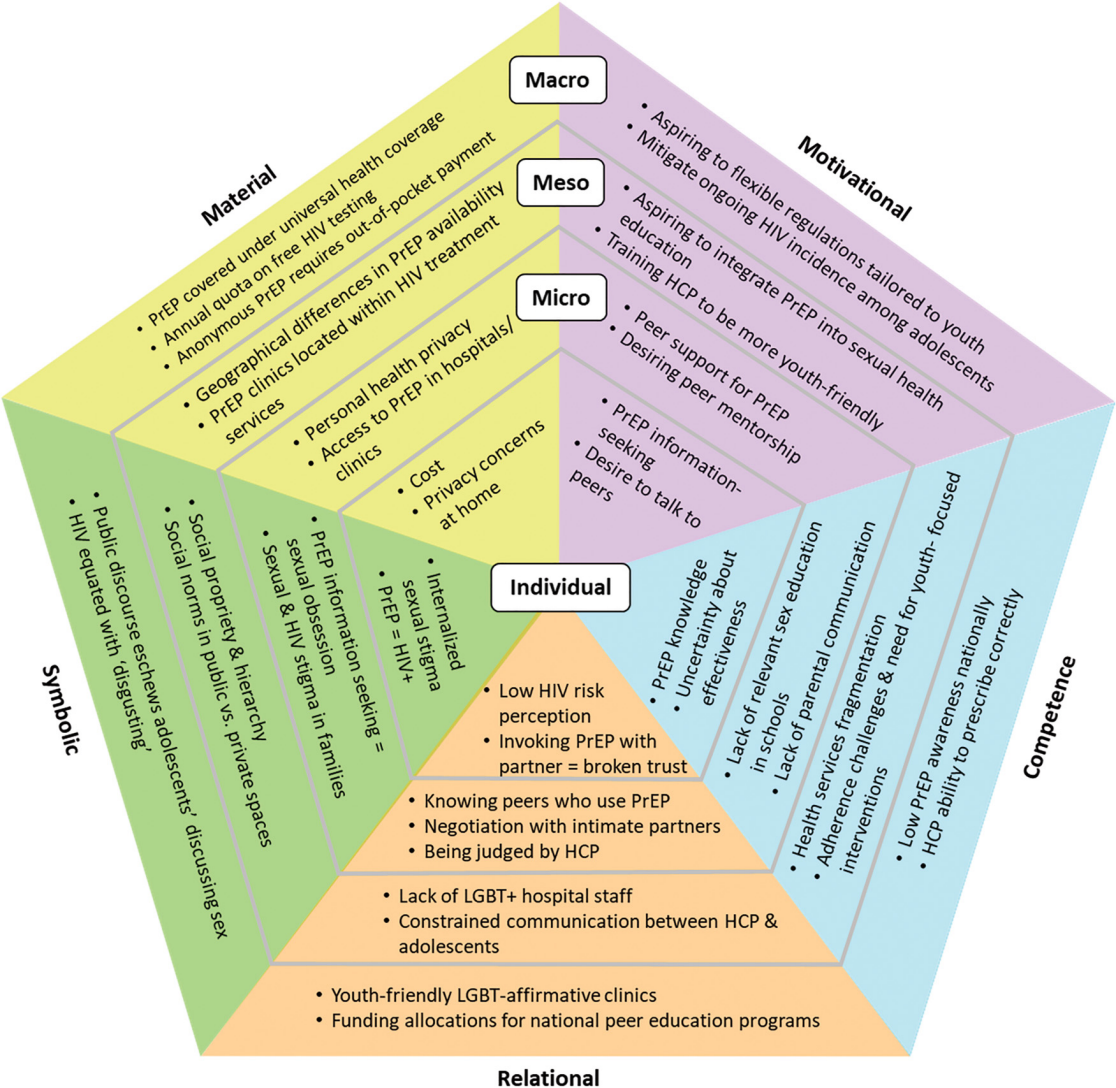
Practice-based factors affecting PrEP engagement at individual, micro, meso, and macro levels among young gay men and young transgender women in Thailand. Abbreviation: PrEP, pre-exposure prophylaxis.

Across FGDs and KI interviews, we identified and categorized findings at each level of young people's social ecology by types of factors that affect engagement with PrEP (see Figure 1).

#### Individual Level

Material factors at the individual level encompassed cost and privacy concerns. As KIs explained, “…PrEP is great if you have money…” (KI-13, manager, NGO); and “They are just in high school, and don’t have much money like people who have jobs; so PrEP cost is an issue for them” (KI-1, director, CBO). Adolescents described concerns that their parents would find out about their PrEP use, thereby revealing their sexual orientation or gender identity: “I don’t dare to be myself at home…I’m afraid that they [my parents] couldn’t accept it” (FGD-2, P-2, YGM). Adolescents’ motivations to learn about PrEP emerged in information-seeking, primarily through digital media: “…I like reading online from many websites; I also watched media shows that talked about PrEP” (FGD-4, P-4, YGM). This mitigated barriers to adolescents’ discussing sex with adults: “If they want to search for it, there is no cultural barrier to this. It is easily accessible…” (KI-7, co-director, CBO).

Competence factors arose in adolescents’ individual knowledge and uncertainties about PrEP, as in the following interchange (FGD-1):

Facilitator (F): …have you heard of or do you know about PrEP?

Participant (P)-6 (YGM): I know a little about it; I’ve seen it on the Internet.

P-4 (YGM): It is like a medicine for people who are not infected to take it for prevention.

P-6 (YGM): It is protection before having sex.

P-5 (YGM): I know of it, but I don’t know how it is taken before or after.

Young people further indicated uncertainties about the effectiveness of PrEP:

P-1 (YTGW): Using a condom is better than getting PrEP because we don’t know if that medicine can actually protect us.

P-6 (YGM): From the webpage, it is mostly about prevention…medicine to be taken before having sex; but I’m not sure it can really help.

Relational factors intersected with (mis)understanding of risk, as well as posing barriers to engaging with healthcare: “They feel normal and fine, so they don’t access healthcare…They think that their partners are good looking and also young, so there will be not any problems about that [HIV]…” (KI-5, nurse, university clinic). Internalized and enacted stigma also emerged as a barrier to accessing prevention: “Firstly, they [MSM and TGW] are socially stigmatized. They are now more accepted, but how much more?…they are still afraid to access the hospital…” (KI-4, nurse, government hospital).

Symbolic concerns at the individual level emerged in intersectional sexual and HIV stigma that deterred PrEP engagement. A CBO director explained, “Still, many young people don’t seek HIV testing and other prevention [PrEP and PEP]…it is due to social stigma and discrimination against key populations who are MSM and TGW” (KI-1, director, CBO). PrEP was described as emblematic of HIV: “…They fear that others will find out, including that they take PrEP. PrEP is the same as ARVs, and that is for HIV treatment…even though PrEP is for prevention…” (KI-5, nurse, university clinic).

#### Micro Level

Privacy concerns due to material challenges in healthcare settings intersected with adolescents’ individual-level concerns. Long wait times along with co-location of PrEP services in HIV treatment clinics amplified adolescents’ privacy concerns, although few HCPs outside of these clinics were reported to be familiar with PrEP. An administrator in a government clinic confirmed, “PrEP is free when you show your Thai ID card” and “…if you don’t want to show your ID, you could anonymously buy it” (KI-19, supervisor, sexual health clinic); however, most adolescents lack the resources to pay out-of-pocket. While private clinics may offer advantages in terms of privacy and ease of access, “if they go to a private hospital or clinic, they have to pay up front; if they want reimbursement, a parent's signature is required for insurance claim documentation” (KI-18, nurse, sexual health clinic). A peer counselor concurred: “…their parents will be informed when there is a medical fee involved” (KI-20, peer counselor, NGO). However, any form of parental notification was described as “…the main barrier that makes young people see no reason to come to these healthcare organizations” (KI-6, physician, government hospital).

Motivations emerged in peer, school, and healthcare settings, with young people and KI peer educators advocating that PrEP be mainstreamed in HIV prevention: “It should be provided all in one package” (FGD-4, P-1, YGM); “It [PrEP] should be introduced to young people as a foundation for them” (KI-16, peer educator, university). A young TGW indicated a desire for mentorship from older TGW peers, “to be able to openly and comfortably talk about everything, like sex…to teach us what we don’t know like use of medication, including anything around hormone use (FGD-3, P6, YTGW). However, service providers expressed a degree of ambivalence: “It [PrEP] is not the right answer. The best way to protect them is to use condoms because there are no side effects…” (KI-9, health counselor, university clinic). Alternately, a physician described the untapped potential for PrEP to support HIV testing motivations: “PrEP can be an important tool to attract people to get an HIV test…people seem to go for HIV testing only when they think they might be infected…not for the purpose of prevention preparation” (KI-6, physician, government hospital).

Competency factors emerged in peer, family, school, and healthcare settings. A peer education program manager explained, “…in trying to understand what it means to be gay and be young, we have to be taught in a way, in school or within our social life” (KI-13, manager, NGO). However, schools largely fail to provide this information: “…obviously sexuality education they get in school is not likely to focus much on sex between men…” (KI-3, lecturer, university). Similarly, “Thai families are rarely open for the parents to talk about sex with their adult children” (KI-15, peer educator, NGO). However, a nurse at a youth clinic described fostering adolescent-parent communication: “My work is not only with youth but also with their parents. I promote understanding for parents about how to react to their teenage children having condoms…we need to include parents in projects for youth” (KI-11, nurse, youth clinic).

Relational factors emerged in young people's invoking the significance of knowing peers who use PrEP: “I have never seen any of my friends or people who are close to me use it” (FGD-1, P-1, YTGW). However, even introducing discussion of PrEP was described as “creat[ing] negativity for them in a relationship…‘What have you done? What happened to you? If you love and trust me, why do we have to use condoms?’” (KI-10, peer educator, NGO). A youth clinic counselor recounted, “At our hospital, a couple came to get PrEP because one person was positive, the other negative. If they didn’t have a conversation or use protection, this might lead to infection because of their trust” (KI-9, health counselor, university clinic). An HCP explained further, “…People who are on PrEP have to hide it because their partners can misunderstand and think that taking PrEP means having HIV” (KI-5, nurse, university clinic).

Family systems also presented relational barriers to PrEP engagement. A young person explained, “If they must let their parents know beforehand, they would not dare do the test” (FGD-1, P-4, YGM). A peer educator corroborated this account: “…When their children are at high risk, very few parents would bring their children here [NGO clinic]. Mostly, the young people find information themselves before walking in here” (KI-15, peer educator, NGO). A physician described intersections between familial and school microsystems:

It is pretty clear that their parents were not taught about sex education, gender, and sexual health. …The parents are not cultural conservatives, but *they* didn’t learn how to use protection; their children…do not get to access information by talking with their parents. (KI-6, physician, government hospital)

Relational challenges between HCPs and adolescents were described as a barrier to sexual healthcare:

…When young MSM and TG go to see a doctor…they don’t dare report their actual symptoms, like having a sore in the anus, because of the [doctor's] facial expression, and way of questioning; it doesn’t help them to be able to speak the truth. (KI-10, peer educator, NGO)

Symbolic factors imbued even seeking out information about HIV prevention with stigma: “Most people still see it [HIV] as something embarrassing or shameful…If we tried to seek information on this, it would be perceived as an obsession…just like pornography…” (KI-16, peer educator, university). HIV and sexual stigma arose in families, constraining communication:

They always look at their children as being too young…Parents sometimes don’t know about their children's behavior, or sexuality and gender identity…Some TGW dress like men when they get back home…some MSM who don’t want to disclose have to pretend to be straight when they’re at home…(KI-5, nurse, university clinic)

In healthcare settings, HIV stigma loomed large as a barrier to accessing HIV services:

When you walk into a hospital or a center where it is obviously known as a health center for HIV testing and prevention, they will be unconfident and scared—not only that the result would come out positive, but they fear being seen by other people they know. (KI-1, director, CBO)

#### Meso Level

Material challenges at the meso level included HCP, institutional, and geographical variations in the application of PrEP guidelines, and PrEP access. A nurse explained clearly, “We provide PrEP for those who often engage in risk behavior and those who have a partner with a different HIV status” (KI-4, nurse, government hospital); but an NGO administrator opined, “…PrEP is not for everyone; you should be kind of sexually active to even qualify for PrEP” (KI-13, manager, NGO). KIs described geographical variations in access as PrEP services are not available in all areas of the country: “PrEP is accessible…but we have to look at the service sites in this region because hospitals or organizations where they support this medicine are rare” (KI-9, health counselor, university clinic). Access was described as more precarious for immigrants and migrant workers: “They are not able to get free PrEP or HIV treatment; some government hospitals have a very small quota in providing free medications” (KI-18, nurse, sexual health clinic).

Motivations on the part of LGBT + NGO, CBO, and student groups reflected community support for integrating PrEP in HIV prevention for young people: “It [PrEP] should be accessible because it is another way of HIV prevention” (KI-17, peer educator, university); “…Indeed, basic prevention, understanding the modes of transmission, PrEP and PEP are key” (KI-15, peer educator, NGO). A youth network leader explained the importance of further integrating PrEP in sexual health education:

PrEP is there, that's great; but it shouldn't mean that you have to dive right into PrEP, just so that you can avoid everything, not having learned about safe sex. The most important thing is to make sure that they are educated about safe sex. (KI-13, manager, NGO)

Competencies at the meso level included lack of HCPs in government hospitals familiar with PrEP guidelines and service fragmentation. While a youth-friendly clinic provided easy access to HIV counseling and testing, young people had to be referred to an affiliated adult sexually transmitted disease (STD) clinic to access PrEP: “Only the HIV testing is fast-tracked at this [youth-friendly] clinic. We only provide PrEP on certain occasions like World AIDS Day and Valentine's Day” (KI-5, nurse, university clinic). However, an administrator from an LGBT + CBO explained, “It is challenging for gay people; some hospitals do not have a staff-person or nurse who specializes in services for sexually diverse populations” (KI-7, co-director, CBO). An HCP further described the need to develop tailored programs to support adolescents: “Personally, I think it is a must [to discuss PrEP]…we know that young people have low adherence, but we have no intervention to boost their adherence…it can be very difficult to retain them in the system…” (KI-6, physician, government hospital).

Relational factors reflected social rules and interactions within institutions, including lack of HCP competencies with YGM and YTGW, socially conservative HCP attitudes, hierarchical power relationships, and sexual stigma. Moreover:

Thai culture is just like that. It relates to the imbalance in power relations between those who provide services and those who receive them. Some healthcare providers are so conservative that teenagers do not dare to access that. They must be very brave to access healthcare services. (KI-14, director, NGO)

A peer educator described trainings they provide to staff and counselors in healthcare settings:

We are trying our best to minimize the sexual stigma in healthcare settings; we have anonymous systems and transgender woman-friendly services for TG clients, and MSM friendly services for MSM clients. They will not be blamed or judged for their behaviors; we provide social support. (KI-15, peer educator, NGO)

An HCP at a government hospital noted the value of LGBT + CBOs: “There are advantages for them [MSM/TG] to take care of their own community because I see that they have someone who leads them like a mother taking care of children. They have connections inside this community…” (KI-11, nurse, youth clinic).

Symbolic factors at the meso level emerged in HIV stigma, and sociocultural norms that constrain talking about sex:

PrEP is a new technology, and it will be another option for people…but Thai norms are that having HIV is an untold story. They don’t know how to tell their partners. They are scared. They want to protect, but they are unable to negotiate that. (KI-10, peer educator, NGO)

However, a university lecturer explained Thai norms around propriety and hierarchy, rather than sexual taboos per se, as limiting discussions in families and schools:

when you ask this question to Thai people, they will usually tell you that discussion of sexuality is taboo in Thai society. For me, being in the field, it's a bit weird because I talk about sex all the time with Thai colleagues…I think generally it goes to the notion of propriety, the time and place do this sort of thing and not to do things…once kids shouldn’t be having sex, we shouldn’t be talking about explicit things with them either. It's not the time and place, it's too early… It's not that Thailand is the only place where this happens, but there's a certain discourse that forms obstacles. (KI-3, lecturer, university)

As a result, “There is no education about it, and they don’t learn how to use protection” (KI-8, nurse, government hospital); and “The students from school next to the hospital have never accessed the [HIV testing and prevention] service…they fear being seen by others who know them, relatives, and neighbors” (KI-11, nurse, youth clinic).

#### Macro Level

The inclusion of PrEP in universal healthcare coverage (“for ART and PrEP, these are free” [KI-14, director, NGO]) has opened up greater possibilities for adolescents; nevertheless, lack of availability in many areas and annual quotas on lab testing (ie, 2 HIV tests/year), pose material barriers to PrEP engagement. A KI explained, “they all now can individually access PrEP because it is included in sexual and reproductive healthcare” (KI-18, nurse, sexual health clinic); however, adolescents who test HIV-negative may be referred to a different hospital with PrEP available, requiring a new HIV test; and this incurs out-of-pocket payment if their annual quota is expended, which is untenable for most young people. An HCP indicated, “According to the regulations…we give it to all ages here in Thailand,” but questioned “How could this rule become flexible for young people to be able to receive services? Services should focus on benefits for young people” (KI-6, physician, government hospital).

Competency factors arose on a national level in lack of awareness about PrEP and where to access it, as well as the need for programs to support young people's adherence.

I think the channel for getting this [PrEP] is not widespread. It's only known within the field of HIV prevention; others who are not in this field don’t know where to get it, which hospitals. It is a dead end for them…(KI-1, director, CBO)

An HCP who advocates for youth services reported, “…our main concern is about their adherence; it has been evidenced that adherence is not good among people under 18 years” (KI-6, physician, government hospital). To this end, a national NGO director described a peer education and outreach program: “We have a project working with young people age 15 to 19 years. We check their blood and provide PrEP… It is very interesting to try and reach them; peer educators have a positive influence on their thinking” (KI-14, director, NGO). An older adolescent concurred: “We are able to create trust with them, and they can talk with us directly. We are able to take care of younger adolescents and gain their trust” (FGD-1, P-4, YGM).

Relational factors at the macro level arose in challenges in navigating the government healthcare system and the paucity of youth-friendly clinics. As indicated by a youth clinic nurse who described assisting young people in navigating an affiliated hospital to access PrEP, “There are barriers for young people…” (KI-11, nurse, youth clinic). A physician's recommendation for systemic changes focused on scaling up youth-friendly clinics: “…If we are able to create healthcare settings where healthcare providers are just like their peers who make young people feel comfortable, this would enhance young people's access to healthcare services, and maybe they’ll bring some friends” (KI-6, physician, government hospital). A nurse described the potential of youth-friendly clinics, as well as concerns in government hospitals: “…Our public health just launched some youth-friendly clinics…we provide them fast-track service…from screening, they are referred to the ‘Teenage Clinic’…But we have to ask, will they come?” (KI-4, nurse, government hospital).

Symbolic factors were described at the macro level in public discourse that invoked stigma in young people's engaging with PrEP: “Thai culture affects that…When people are having sex, the culture already places them in the frame…Thai culture and context limit access to prevention tools among young people” (KI-9, health counselor, university clinic). However, a CBO director described spaces that allow for public education and representation of HIV prevention, though largely circumscribed to major urban areas:

In major metropolitan areas, and I am talking about large cities, like Bangkok, like Pattaya…there are more opportunities for public information and communication. I went to the Bangkok Art and Culture Centre…there was a kiosk set up by prevention groups who were distributing condoms and information about HIV prevention. And that's a public setting where a lot of gay youth might go…(KI-2, director, CBO)

## Discussion

This study with gay and transgender adolescents, and key informant HCP and CBO/NGO staff working with young key populations, reveals broad support and increasing potential for the integration of PrEP into HIV prevention and sexual health programs for young people in Thailand. However, while the inclusion of PrEP in universal healthcare coverage and the absence of lower age limits for use represent substantial macrosocial interventions to enable PrEP use among adolescents—many of whom otherwise would not be able to afford it—we identified systemic barriers at microsocial, mesosocial, and macrosocial levels that explain the very low uptake of PrEP among young people in Thailand, as well as identifying strategic pathways to promote PrEP engagement.

Using a practice-based framework, our analysis highlights how similar factors (ie, relational) enacted in multiple complementary spheres of influence (ie, family, school, and healthcare systems) create systemic barriers to adolescents’ engagement with PrEP. For example, expectable relational gaps in communication about sex and sexuality between sexual and gender minority adolescents and their parents are replicated in their experiences in schools (no MSM-relevant or TGW-relevant sexual health education) and healthcare settings (fraught communication with judgmental and stigmatizing HCPs). PrEP awareness and engagement is in effect relegated to individual young people's motivations and competencies to explore social media, to locate and engage with LGBT + CBOs (if locally available), and to navigate government healthcare clinics often experienced as unfriendly environments. In an earlier cross-sectional survey of at-risk YMSM and YTGW (n = 260) in Thailand, 43% reported HCPs exhibited hostility toward them, and 31% being given less attention than other patients.^34^

The practice-based framework also illustrates multilevel factors that intersect to constrain opportunities for PrEP engagement, despite the existence of public health regulations on the macro level that have expanded PrEP coverage to include adolescents >15 years old. These include geographical variations in PrEP availability, longer timelines to receive PrEP in government versus other facilities, having to access PrEP in HIV treatment clinics and from HCPs with varying competencies and sometimes stigmatizing attitudes toward YGM and YTGW, and lack of awareness of the availability of free PrEP.^8,35^ This renders the already challenging prospect of accessing PrEP—that adolescents must navigate a healthcare system designed for adults—even more challenging given the unpredictability of the healthcare settings and HCPs they might encounter. Several HCPs also emerged as champions of youth empowerment and decision making, nevertheless, themselves having to navigate otherwise disenfranchising institutional practices.

What remains predictable are multilevel symbolic representations of PrEP, more so among adolescents. Thailand is not socially or culturally unique in the stigma surrounding PrEP use^36,37^; however, internalized and enacted stigma among MSM and TGW in Thailand^27,38^ may be amplified in a system that is imbued with powerful norms about propriety (eg, how, when, and with whom adolescents can talk about sex and HIV among adults) and deferring one's private individual needs to maintain public social harmony (with parents, teachers, HCPs).^39^ PrEP becomes emblematic of social and cultural impropriety: if adolescents shouldn’t talk with adults about sex or be known to engage in sex, PrEP access requires defiance of sociocultural norms—the antithesis of an enabling environment. In addition, PrEP-related stigma was evidenced in families, schools, and in healthcare settings among HCPs otherwise charged with supporting health and prevention, and intersected with sexual-, gender nonconformity-, and HIV-stigma, similar to intersectional PrEP stigma described among racialized youth in the United States.^36^ PrEP stigma was also evidenced in adolescents’ intimate relationships, with PrEP signifying mistrust; while not unique to MSM partners in Thailand,^40,41^ PrEP stigma may be animated by values that privilege relational harmony over individual needs.^32^

In addition to elucidating the network of factors that constrain adolescents’ engagement with PrEP, our multilevel practice-based analysis suggests directions for programmatic initiatives, policy advocacy, and research to strategically position PrEP as a viable prevention tool for gay and transgender adolescents in Thailand. Table 2 illustrates select interventions that emerged from our findings and their multilevel impacts on PrEP engagement.

**Table 2.** Select Interventions, Practice-Based Factors Across Social Ecological Levels, and Target Outcomes to Facilitate PrEP Engagement Among Young Gay Men and Young Transgender Women in Thailand.

Select interventions	Social ecological level				Target outcomes
	Individual	Micro	Meso	Macro	
Youth-friendly gay-affirming and gender-affirming clinics	*Motivational*				Facilitate desire to seek healthcare through affirming spaces, including provision of PrEP in gender-affirming clinics that also provide hormone therapy
		*Relational*			Promote positive interactions through differentiated service delivery of gay-affirming and gender-affirmative care
			*Symbolic*		Combat intersectional stigma in healthcare settings
				*Material*	Allocate public health funding to scale up both gay-affirming and gender-affirming youth-friendly clinics, and respect confidentiality
Tailored interventions to support PrEP adherence	*Motivational*				Promote self-efficacy in controlling HIV risk
		*Competence*			Train and support peer adherence counselors
			*Competence*		Test and evaluate mHealth apps to support adherence
				*Material*	Ensure consistent PrEP availability in CBOs and government clinics; reduce waiting times to access PrEP
PrEP-focused/HIV prevention clinics in CBOs and government hospitals	*Symbolic*				Avert conflation of PrEP and ART, and mitigate PrEP stigma
		*Competence*			Train HCPs, including in CBOs, to implement PrEP
			*Material*		Reduce service fragmentation and unavailability of HCPs to provide PrEP
				*Motivational*	Marshal national efforts to scale-up PrEP and meet UNAIDS targets of no new HIV infections by 2030
Peer education programs	*Competence*				Promote adolescents’ PrEP knowledge and awareness
		*Relational*			Forge relationships with PE organizations
			*Competence*		Incorporate the expertise of gay and transgender youth in designing PrEP education and service provision
				*Symbolic*	Mitigate sociocultural proscriptions against adolescents talking to adults about sex

Abbreviations: ART, antiretroviral treatment; CBOs, community-based organizations; HCPs, healthcare providers; HIV, human immunodeficiency virus; PE, peer education; PrEP, pre-exposure prophylaxis.

Youth-friendly clinics are a well-supported strategy to engage adolescents in healthcare that is experienced as inviting and affirming^42,43^—supporting individual motivations to seek care through effecting positive relational factors at the microsocial level, as well as symbolizing acceptance—the antithesis of stigma. Allocation of government public health funding to scale up youth-friendly clinics, train and support HCPs, and evaluate impacts on PrEP use and HIV infections averted may be a cost-effective macrosocial intervention.^44^ Nevertheless, suboptimal PrEP implementation and adherence are associated with multilevel barriers, as among young people in other settings,^36,45,46^ with documented lower adherence among younger MSM and TGW than those >25 years, and among TGW versus MSM in Thailand.^47,48^ This supports the need for tailored, youth-focused programs to facilitate PrEP adherence.^4,14^ Differentiated service delivery of PrEP through youth-friendly clinics with peer educator involvement should include trans-specific spaces that provide gender-affirming care and integrate PrEP with hormone therapy and other sexual health services,^49–51^ such as the Tangerine Community Health Clinic in Bangkok,^6^ as well as gay-affirmative clinics serving YMSM.

Peer education by and for sexual and gender minority adolescents may function on multiple levels: by displacing sociocultural proscriptions (ie, symbolic) at the macro level that constrain young people's communication with adults about sex and HIV, by forging relational ties at the microsocial level between young people and peer education organizations, and by promoting individual competencies and motivations through PrEP education and awareness.^42^ Peer navigators can also assist young people in engaging with complex healthcare institutions,^52^ with peer organizations collaborating with NGOs and CBOs to advocate shifts in institutional norms to promote youth-friendly practices, such as ensuring thorough implementation of minor consent regulations to protect adolescents’ confidentiality in accessing PrEP.^53^

Finally, a practice-based HIV prevention framework illustrates the counterproductive impacts of Thai government regulations announced in October 2022, which discontinue government funding to reimburse civil society groups for the costs of PrEP and HIV testing of individuals not covered by Universal Health Care*.*^10,54^ With an estimated 60% to 70% of PrEP in Thailand provided through civil society organizations—including the preponderance of gay and transgender people who choose to avoid discrimination and stigma from HCPs in government clinics despite having Universal Health Coverage—this policy reversal in effect discontinues coverage for the majority of people currently receiving PrEP.^54^ It is also antithetical to a key population-led health services model, which has been responsible for successes in scaling up PrEP and HIV service delivery in Thailand.^11,55^ In the case of young gay and transgender people, the impact of constraining PrEP access to institutional settings, such as government hospital clinics, reverberates across social ecological levels, and runs counter to the recommended interventions presented in Table 2. Practice-based impacts can be expected in reduced motivations among young people to seek PrEP, HCPs with lesser competencies to provide PrEP in the context of gay-affirming and gender-affirming care, and material constraints in constricted geographical availability of PrEP and increased bureaucratic hurdles, wait times, and threats to young people's privacy and confidentiality.

### Strengths and Limitations

As a qualitative study, results cannot be generalized to other YMSM and YTGW across Thailand; however, we purposefully engaged youth outside major urban centers, which are the focus of most HIV research. Our successful involvement of 16-year-olds to 20-year-olds, who are often left out of PrEP research including in the Southeast Asian context, is aligned with UNAIDS best practices to support differentiated service delivery for adolescents.^12,18^ Despite restrictive dress codes regulating clothing and hair length, which make it difficult for transgender young people to express their gender identity in school settings,^56^ we successfully engaged several transfeminine youths. However, the small number of young people who identified as transgender limits our ability to address the breadth of concerns about PrEP use across the spectrum of transgender and gender diverse youth, including transmasculine and gender nonbinary young people. Perspectives and experiences of YTGW and YGM study participants were corroborated by sexually and gender diverse adult KIs from across Thailand, including those working in trans-specific settings.

The study time frame amid several PrEP demonstration projects and evolving government policies that include PrEP in universal healthcare coverage may not fully reflect the current PrEP scenario in Thailand. However, Thai government data document substantial and enduring gaps in PrEP coverage among key populations.^8^ We also conducted interviews with KIs at 2 time points, including individuals well-positioned to identify institutional changes in response to evolving government regulations, who corroborated ongoing challenges for PrEP engagement among young people. Finally, we did not explore the acceptability of long-acting injectable (LAI)-PrEP, which may mitigate some of the challenges in adherence to daily pills or on-demand PrEP among adolescents^14,57^; however, the 5-year trajectory from initial licensure to government coverage of oral PrEP suggests that LAI-PrEP may not be accessible in Thailand for several years. Our findings suggest that LAI-PrEP may be an advantageous option for adolescents at risk—however, they also indicate caution in approaching this new technology as a biomedical prevention tool decontextualized from young people's experiences and local ecologies.^58,59^

## Conclusions

Results of this study transpose approaches to PrEP as a biomedical prevention tool to be added to combination prevention to instead foreground public health policies, institutional and HCP practices and competencies, family and school environments, and pervasive HIV and sexual stigma, which produce systemic barriers to adolescents’ engagement with PrEP. As a result, gay and transgender adolescents are in effect required to defy sociocultural norms, navigate complex and fragmented healthcare systems designed for adults, risk censure from HCPs, teachers, and parents, and seek relevant sexual health information on their own in order to engage with PrEP. Differentiated service delivery of PrEP for adolescents in Thailand would benefit from meaningfully involving sexually and gender diverse adolescents in the development and scaling up of youth-friendly, gay-affirming and gender-affirming community clinics, PrEP dosing options and adherence supports, peer education programs, national campaigns to combat HIV and sexual stigma, as well as advocacy for regulatory changes to mitigate privacy and confidentiality risks in government healthcare and insurance systems. Tailored programs to integrate PrEP into combination HIV prevention and comprehensive sexuality education for young key populations are vital to attaining 2030 targets of no new HIV infections in Thailand.
